# MCT1 in Invasive Ductal Carcinoma: Monocarboxylate Metabolism and Aggressive Breast Cancer

**DOI:** 10.3389/fcell.2017.00027

**Published:** 2017-04-03

**Authors:** Jennifer M. Johnson, Paolo Cotzia, Roberto Fratamico, Lekha Mikkilineni, Jason Chen, Daniele Colombo, Mehri Mollaee, Diana Whitaker-Menezes, Marina Domingo-Vidal, Zhao Lin, Tingting Zhan, Madalina Tuluc, Juan Palazzo, Ruth C. Birbe, Ubaldo E. Martinez-Outschoorn

**Affiliations:** ^1^Department of Medical Oncology, Sidney Kimmel Cancer Center, Thomas Jefferson UniversityPhiladelphia, PA, USA; ^2^Department of Pathology, Memorial Sloan Kettering Cancer CenterNew York, NY, USA; ^3^Medical Oncology, National Cancer InstituteBethesda, MD, USA; ^4^Pathology, University of Rome Tor VergataRome, Italy; ^5^Department of Pathology, Anatomy and Cell Biology, Thomas Jefferson UniversityPhiladelphia, PA, USA; ^6^Division of Biostatistics, Department of Pharmacology and Experimental Therapeutics, Thomas Jefferson UniversityPhiladelphia, PA, USA; ^7^Department of Pathology, Cooper University HospitalCamden, NJ, USA

**Keywords:** triple negative breast cancer, glycolysis, oxidative phosphorylation, lactic acid, tumor microenvironment

## Abstract

**Introduction:** Monocarboxylate transporter 1 (MCT1) is an importer of monocarboxylates such as lactate and pyruvate and a marker of mitochondrial metabolism. MCT1 is highly expressed in a subgroup of cancer cells to allow for catabolite uptake from the tumor microenvironment to support mitochondrial metabolism. We studied the protein expression of MCT1 in a broad group of breast invasive ductal carcinoma specimens to determine its association with breast cancer subtypes and outcomes.

**Methods:** MCT1 expression was evaluated by immunohistochemistry on tissue micro-arrays (TMA) obtained through our tumor bank. Two hundred and fifty-seven cases were analyzed: 180 cases were estrogen receptor and/or progesterone receptor positive (ER+ and/or PR+), 62 cases were human epidermal growth factor receptor 2 positive (HER2+), and 56 cases were triple negative breast cancers (TNBC). MCT1 expression was quantified by digital pathology with Aperio software. The intensity of the staining was measured on a continuous scale (0-black to 255-bright white) using a co-localization algorithm. Statistical analysis was performed using a linear mixed model.

**Results:** High MCT1 expression was more commonly found in TNBC compared to ER+ and/or PR+ and compared to HER-2+ (*p* < 0.001). Tumors with an *in-situ* component were less likely to stain strongly for MCT1 (*p* < 0.05). High nuclear grade was associated with higher MCT1 staining (*p* < 0.01). Higher T stage tumors were noted to have a higher expression of MCT1 (*p* < 0.05). High MCT1 staining in cancer cells was associated with shorter progression free survival, increased risk of recurrence, and larger size independent of TNBC status (*p* < 0.05).

**Conclusion:** MCT1 expression, which is a marker of high catabolite uptake and mitochondrial metabolism, is associated with recurrence in breast invasive ductal carcinoma. MCT1 expression as quantified with digital image analysis may be useful as a prognostic biomarker and to design clinical trials using MCT1 inhibitors.

## Introduction

Breast cancer remains the most common cancer diagnosed and the second most common cause of cancer-related death in US women in 2016 despite advances in early detection and novel treatments (Siegel et al., 2016). Risk-stratification of breast cancer is predominantly based on the presence or absence of the hormone receptors for estrogen (ER) and progesterone (PR), overexpression of human epidermal growth factor receptor 2 (HER2), clinical staging, and in some cases, selected gene expression profiles. ER and HER2 are both prognostic biomarkers and are used to predict response to antiestrogen drugs and HER2 inhibitors.

Human breast cancer has a different metabolic rate compared to normal breast tissue. Tumors frequently have very high levels of lactate in their microenvironment, produced by aerobic glycolysis. Otto Warburg hypothesized that cancer cells contained dysfunctional mitochondria leading to their inability to utilize oxidative phosphorylation to generate ATP and forcing them to use aerobic glycolysis (Koppenol et al., 2011). We now know that in many cases of human breast cancer, mitochondria are not dysfunctional and in fact some cancer cells have very high mitochondrial oxidative phosphorylation (OXPHOS) (Martinez-Outschoorn et al., 2017). Glycolysis is more energetically inefficient compared to OXPHOS and why cells within tumors would utilize glycolysis has remained a paradox (Vander Heiden et al., 2009).

Detailed characterization of breast tumor metabolism has revealed that there is intratumoral metabolic heterogeneity with some cells being glycolytic and generating lactic acid, while others have high mitochondrial metabolism. Metabolic heterogeneity might explain the apparent tumor metabolism paradox since it increases energetic efficiency (Martinez-Outschoorn et al., 2017). Metabolic heterogeneity can be induced in experimental models of breast cancer by oxidative stress, which damages the mitochondria of intratumoral stromal cells and induces a metabolic switch to aerobic glycolysis (Pavlides et al., 2009; Martinez-Outschoorn et al., 2012). Conversely high antioxidant activity via activation of the pentose phosphate pathway in a subgroup of carcinoma cells allows these cells to maintain high mitochondrial oxidative phosphorylation metabolism (OXPHOS) (Ko et al., 2016). Lactate is one of the links between these two intratumoral compartments since it can be produced by glycolytic tumor stromal cells and then taken up by carcinoma cells to be utilized for mitochondrial OXPHOS and ATP production (DeNicola and Cantley, 2015).

Monocarboxylate transporters (MCTs) play a key role in this symbiotic relationship between carcinoma cells and other cells of the tumor microenvironment since they regulate the release and uptake of lactic acid and the extracellular pH of the tumor (Martinez-Outschoorn et al., 2017). Metabolic reprogramming of the cancer stroma may provide a compensatory mechanism for cancer cells to survive in an energetically efficient manner in the harsh tumor environment. Lactate is mainly taken up by cancer and non-cancer cells via monocarboxylate transporter 1 (MCT1) (Pinheiro et al., 2010a, 2012; Jones and Morris, 2016). Cancer cells frequently express MCT1 (Peiris-Pages et al., 2016).

MCT1 has been correlated with increased disease aggressiveness across various solid malignancies. Previous work has shown that MCT1 is expressed in human breast, ovarian, cervical, lung, and colorectal cancers, highlighting its importance as a potential marker and therapeutic target across multiple tumor types (Pinheiro et al., 2010b). Pinhero et al. have also specifically showed an increase in MCT1 expression in basal-like breast cancer (BLBC) (Pinheiro et al., 2010a). Cytokeratin 5 is positive in BLBC and the majority of BLBC are triple negative breast cancers since they are negative for ER, PR, and HER2 (Fadare and Tavassoli, 2008).

MCT1 is associated with aggressive disease in models of breast, gastrointestinal, and squamous cell carcinomas (Koukourakis et al., 2006; Bonuccelli et al., 2010; Pinheiro et al., 2010a; Martinez-Outschoorn et al., 2011). In breast cancer models, fibroblasts surrounding malignant cells demonstrate low caveolin 1 expression, a loss which enhances aerobic glycolysis in these cells, with concurrent increased mitochondrial activity and high expression of MCT1 transporter in the epithelial cancer cells with uptake of catabolites (Bonuccelli et al., 2010). In a study carried out by Oliveira et al. expression of MCT1 and MCT4 was present in 90% of GISTs, findings concordant with the high degree of glycolytic metabolism in these tumor types (de Oliveira et al., 2012). Also, MCT1 was found in both the carcinoma cell compartment as well as proliferating basal stem cells in head and neck cancers, underscoring its importance in cell proliferation (Curry et al., 2013).

We hypothesized that altered tumor metabolism with high monocarboxylate uptake in carcinoma cells is a feature of aggressive breast cancers and that higher MCT1 expression will be found in cancer cells of this clinical subtype. This is in keeping with the observations of others that MCT1 is expressed in basal-like breast cancer. To evaluate this hypothesis, we stained breast cancer tissue microarrays totaling 257 patients for MCT1 and digitally analyzed the expression patterns. We have furthermore investigated the value of MCT1 as a prognostic marker in this cohort.

## Materials and methods

### Subjects

The Institutional Review Board at Thomas Jefferson University approved the protocol for this study. Samples of breast cancer were obtained from 257 subjects at Thomas Jefferson University Hospital. Patient data were collected including age, sex, staging by AJCC Version 7 criteria, size of the primary tumor, number of positive lymph nodes, grade, histologic subtypes, mitotic index, Ki67, lymphovascular invasion, resection margins (if applicable), treatment including the use of chemotherapy, radiation therapy, and hormonal therapy, recurrence, and vital status (Edge et al., 2010).

With respect to hormone receptor status, >1% of cells positive by immunohistochemistry were consider to be positive for both estrogen receptor and progesterone receptor status. For HER2 status, the current guidelines of 3+ staining by immunohistochemistry with >30% of invasive tumor cells showing staining, ISH positive based on single probe average HER2 copy number of > = 6 signals/cell, or ISH positive based on dual probe HER2 to CEP17 ratio > = 2.0 were used as cut-offs.

### Samples and immunohistochemistry

A total of 532 human samples of breast carcinoma were studied to evaluate the metabolism of cancer cells within the tumor samples representative of 257 distinct patients. When possible, samples were run in duplicate or triplicate based on the amount of tissue present in the remaining pathology specimens.

Samples were stained by immunohistochemistry for MCT1. All cancer present on a slide and its dominant staining pattern were considered when determining the percent of immune-positive cancer cells in a sample. Human tissues for analysis were fixed in neutral buffered formalin and then embedded in paraffin. Sections (4 μm) were dewaxed, rehydrated through graded ethanols, and antigen retrieval was performed on the Ventana Discovery ULTRA staining platform using Discovery CCI (Ventana cat#950-500) for a total application time of 64 min, followed by MCT1 antibody incubation for 45 min. Secondary immunostaining used a Horseradish Peroxidase (HRP) multimer cocktail (Ventana cat#760-500) and immune complexes were visualized using the ultraView Universal DAB (diaminobenzidine tetrahydrochloride) Detection Kit (Ventana cat#760-500). Slides were then washed with a Tris based reaction buffer (Ventana cat#950-300) and stained with Hematoxylin II (Ventana cat #790-2208) for 8 min.

Quantitative analysis of MCT1 was also performed employing digital pathology with Aperio software. Tissue sections were scanned on a ScanScope™ XT with an average scan time of 120 s (compression quality 70). Images were analyzed using the Color Deconvolution, the Colocalization, and the Membrane Aperio Image Analysis tool. For the Color Deconvolution and Colocalization, analysis areas of staining were color separated from hematoxylin counter-stained sections and the intensity of the staining was measured on a continuous scale from 0 (black) to 255 (bright white). For the membrane analysis, the algorithm detects the membrane staining for the individual tumor cells in the selected regions and quantifies the intensity and completeness of the membrane staining. Tumor cells are individually classified as 0, 1+, 2+, and 3+ based on their membrane staining intensity and completeness. A tumor cell is classified 1+ when there is only partial membrane staining or weak membrane staining. A tumor cell is classified 2+ when there is moderate and complete membrane staining. A tumor cell is classified 3+ when there is intense and complete membrane staining. For each sample the whole tumor area was analyzed. Tumor specimens were considered “positive” for MCT1, when greater than or equal to 30% of the cells analyzed stained at an intensity of 2+ or greater, as previously published (Curry et al., 2013).

### Statistical methods

The expression of MCT1 in human breast specimens was determined as above. Associations between estrogen receptor, progesterone receptor, and HER2 expression and the membrane expression of MCT1 defined as the percentage of cells analyzed expressing 2+ or greater stain intensity were performed using multivariate linear regression with adjustment on heteroskedasticity. Associations of MCT1 expression with race, menopausal status, histologic and nuclear grade, mitotic score, histologic subtypes, Ki67 scoring, tumor size, and the presence of lymphovascular invasion were performed using simple linear regression analysis. Hazard ratios for both risk of recurrence and overall survival were performed using multivariate cox proportional hazard ratios.

## Results

### Baseline patient characteristics

Our patients included 257 individuals with a diagnosis of invasive breast cancer treated at Thomas Jefferson University Hospital between the years of 2000 and 2008. Characteristics of these patients are shown in Table 1. The average patient age was 57.2 with a range from 26.9 to 97.8 years. We collected self-identified information on race of the patients, which included 168 white, 66 black, 10 Asian, 3 Hispanic, and 10 samples where the race is unknown. Menopausal status was determined by the report of the patients through review of their medical oncologist's office notes. Of the 99 patients for whom this information was recorded, 22 were premenopausal, 22 were listed as peri-menopausal, and 55 were postmenopausal at the time of their initial diagnosis.

**Table 1:** **Characteristics of the samples included in the breast tissue microarray**.

**Age**	57.2 (26.9–97.8)
**RACE (SELF-IDENTIFIED)**	
White, non-hispanic	168
Black	66
Asian	10
Hispanic	3
Unknown	10
**MENOPAUSAL STATUS**	
Pre-menopausal	22
Peri-menopausal	22
Post-menopausal	55
Unknown	158
**HISTOLOGY**	
IDC	254
ILC	4
LN or Metastasis	7
**STAGE-TUMOR**	
T1	116
T2	95
T3	16
T4	13
Tx	17
**STAGE-NODAL**	
N0	124
N1	98
N2	13
N3	1
Nx	21
**STAGE-METASTASES**	
M0	195
M1	8
Mx	54
**RECEPTOR PROFILE COMPOSITES**	
ER and/or PR+ HER2-	138
ER and/or PR+ HER2+	42
ER and/or PR− HER2+	20
TNBC	56

*IDC, Invasive Ductal Carcinoma; ILD, Invasive Lobular Carcinoma; LN, Lymph Node; ER/PR, Estrogen/Progesterone Receptor; PR, Progesterone Receptor; HER2, Human Epidermal Growth Factor Receptor 2 status*.

With respect to tumor characteristics, 116 patients had T1 tumors, 95 were T2, 16 were T3, 13 were T4, and 17 were unknown. In 124 patients their lymph nodes were negative, 112 were positive, and 21 were unknown. Only 8 patients had metastatic disease at the time of diagnosis. The ER, PR, and HER2 characteristics of the subjects are also provided in Table 1.

### Association with receptor status

Individually, ER, PR, and HER2 positivity were all negatively associated with MCT1 expression. There is an association between ER negative status and high MCT1 expression and between HER2 negative status and high MCT1 expression (*p* < 0.003 and 0.045, respectively). There was a trend between PR negative status and high MCT1 expression but this did not reach the level of statistical significance (*p* < 0.09; Table 2). Thus, we further investigated TNBC (ER negative, PR negative, and HER2 negative tumors) and found an association between high MCT1 expression and TNBC compared to the other subtypes with an estimated difference of 27% (*p* < 0.001; Table 2).

**Table 2:** **Clinico-pathological correlations with MCT1 staining in epithelial cancer cells**.

**Characteristic**	**Estimated difference**	***p*-value**
**RACE (RELATIVE TO WHITE)**		
Black	−0.03	0.875
Asian	0.18	0.169
Hispanic	0.07	0.953
Pre-menopausal vs. post-menopausal	0.12	0.052
ER+	−0.16	0.002
PR+	−0.08	0.089
HER2+	−0.08	0.044
Triple negative	0.27	< 0.001
Size	Odds ratio 0.03	0.002
High mitotic score	0.13	< 0.001
High nuclear grade	0.16	< 0.001
Ki67	0.37	0.715
LVI	−0.01	0.833
**PRESENCE OF HISTOLOGIC SUBTYPES**		
Apocrine	−0.02	0.891
Colloid	−0.06	0.660
Comedo	−0.02	0.685
Cribriform	−0.08	0.092
Lobular	0.09	0.179
Metaplastic	−0.05	0.771
Micropapillary	−0.02	0.815
Mixed	−0.33	0.159
Neuroendocrine	−0.11	0.633
Pleomorphic	0.13	0.275
Solid	−0.11	0.008
Tubular	0.09	0.510
*In situ* component identified in the sample	−0.09	0.043

*ER, estrogen receptor; PR, progesterone receptor; HER2, Human Epidermal Growth Factor Receptor 2; LVI, Lymphovascular Invasion*.

### Correlation of histologic features with MCT1 expression

**Figure 1:**
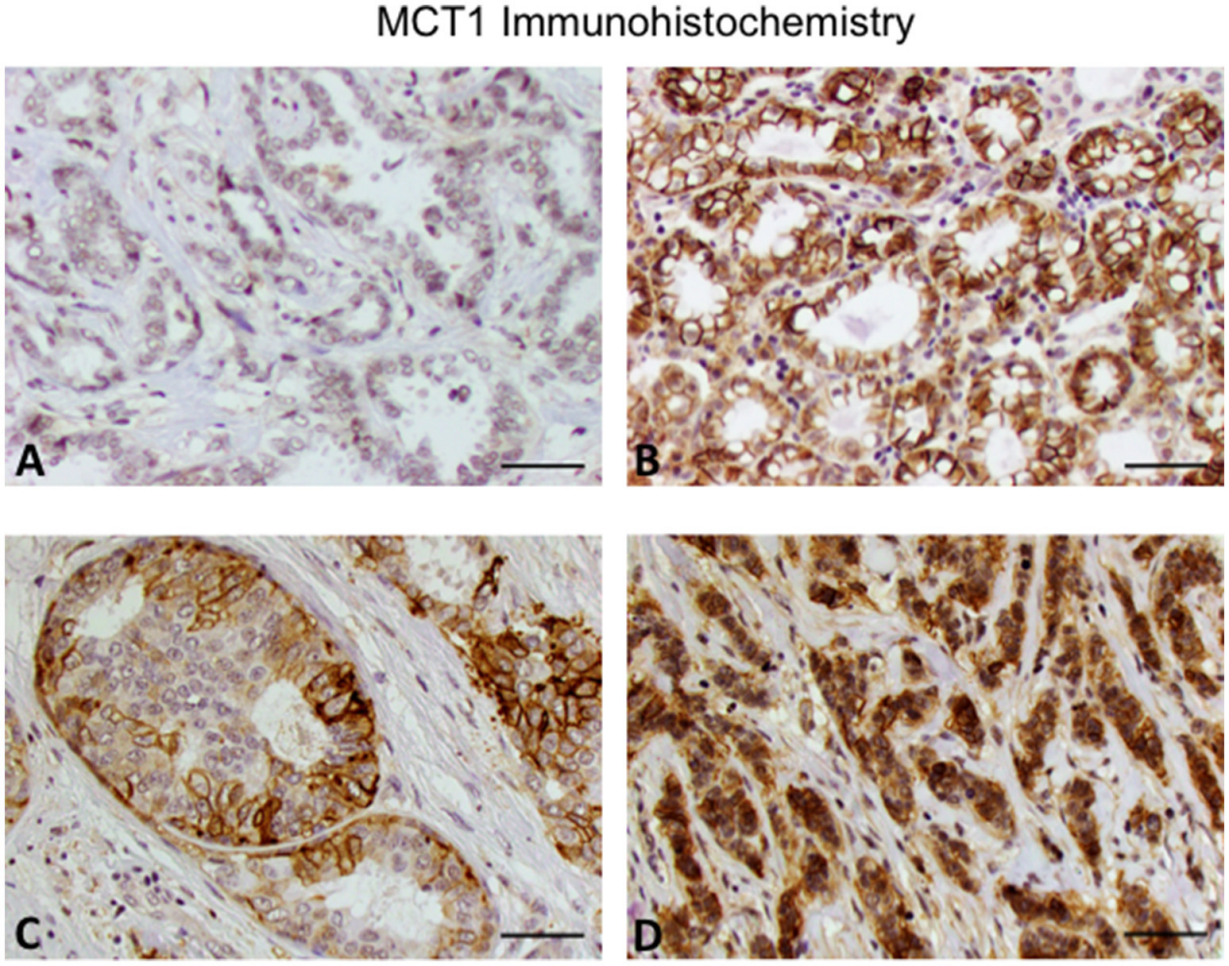
**Expression of MCT1 in invasive breast cancer. (A)** Invasive ductal carcinoma grade 2. **(B)** Invasive ductal carcinoma grade 3. **(C)** Cribriform pattern. **(D)** Invasive lobular carcinoma grade 3. Note that the grade 2 invasive ductal carcinoma has lower MCT1 expression compared to the other subtypes. Original magnification 20x (scale bar 50 μM).

In addition to receptor status, we also investigated other histologic features. Tumors with an identifiable *in situ* component were less likely to stain strongly for MCT1 (*p* = 0.043; Table 2). We therefore also reviewed the presence of different histologic subtypes for potential correlation with MCT1 expression (Figure 1). There was no statistically significant correlation with apocrine features (*p* = 0.891), colloid (*p* = 0.660), comedo (*p* = 0.685), cribriform (*p* = 0.092), lobular (*p* = 0.179), metaplastic (*p* = 0.771), micropapillary (*p* = 0.815), mixed histologies (*p* = 0.159), neuroendocrine (*p* = 0.633), pleomorphic (*p* = 0.13), or tubular subtypes (*p* = 0.510). It is important to note that only a small number of samples had any of these features. In contrast, the presence of a solid subtype was associated with lower MCT1 staining with an estimated difference of 11% (*p* = 0.008, *N* = 82; Table 2).

High nuclear grade, defined as tumor samples scored as a 3 vs. those scored as either 1 or 2 was significantly associated with higher MCT1 staining with a *p*-value of < 0.001. Similarly, a high mitotic score (score of 3 vs. those with scores of 1 and 2) was also associated with higher MCT1 staining (grade 3 vs. grades 1 and 2) with *p* < 0.001. Conversely, the presence of lymphovascular invasion and Ki67 rates scored by the pathologists were not associated with MCT1 expression (*p* = 0.833 and *p* = 0.715, respectively; Table 2).

### Correlation of clinical features with MCT1 expression

We also reviewed patient characteristics with regards to MCT1 expression. These included age, self-identified race, menopausal status, tumor, and lymph node stage by AJCC criteria, and tumor size (Table 2). No statistically significant differences were noted in MCT1 expression patterns between racial groups. However, MCT1 staining was 12% higher in premenopausal women's tumors as compared to their post-menopausal counterparts (*p* < 0.053). Younger age was statistically associated with higher MCT1 staining *p* < 0.001 but the magnitude of this difference was estimated at only 3% with a confidence interval from 1 to 5%. Thus, this is likely of little clinical significance.

Comparisons were made both by individually comparing T1a, 1b, 1c, 2, 3, 4a, 4b, and 4d and by comparing all T1, 2, 3, and 4 samples. Higher T stage tumors were noted to have a higher expression of MCT1, as summarized in Table 3. Tumor size, considered as a continuous variable, was also positively associated with MCT1 expression with an odds ratio of 0.03 and *p*-value of 0.002 (Table 2). Nodal status was not associated with MCT1 expression.

**Table 3:** **T stage correlation with MCT1 staining**.

**AJCC Clinical Stage**	**vs. T1**	**vs. T2**	**vs. T3**	**vs. T4**
**T1**		−0.05 (*p* < 0.18)	0.19 (*p* < 0.001)	0.21 (*p* < 0.001)
**T2**			0.14 (*p* < 0.04)	0.16 (*p* < 0.02)
**T3**				0.02 (*p* < 0.99)

### Outcome correlations data

**Figure 2:**
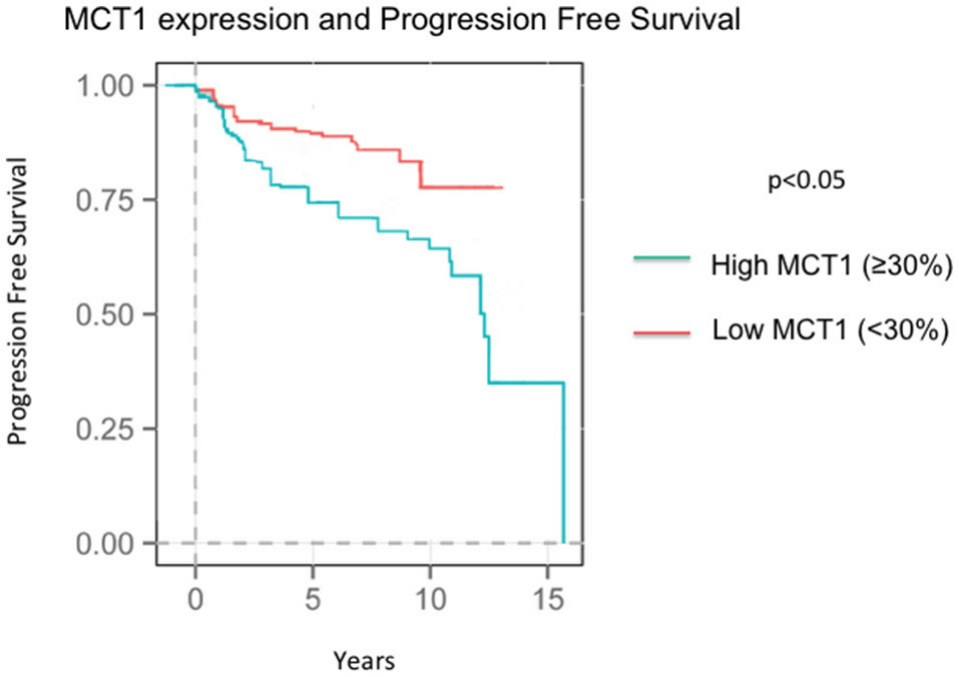
**Progression Free Survival in patients expressing high MCT1 (≥30%) and low MCT1 (<30%)**.

At the time of the collection of our data, survival data was available with an average follow up time of 8.04 years. Seventy-three patients recurred by the time data was collected at an average of 4.37 years after diagnosis. Higher MCT1 staining was predictive of recurrence with a hazard ratio of 2.82 and a *p*-value of 0.024 (Table 4, Figure 2). Of the 257 patients presented here, 196 were alive at the time of data analysis. The overall survival of the sum of all patients was 5.68 years. MCT1 staining trended toward an association with overall survival but this was not statistically significant, with a hazard ratio of 1.89 and *p*- value of 0.171 (Table 4).

**Table 4:** **Correlation of MCT1 staining with recurrence and overall survival using a Cox Regression Model**.

**Outcome measure**	**Hazard ratio**	***p*-value**
Recurrence	2.82	0.024
Overall survival	1.89	0.171

## Discussion

### Triple negative breast cancer is associated with higher expression of MCT1

Our work has shown a statistically significant association between ER negative, HER2-negative, and triple negative breast cancer and high MCT1 expression. This is in keeping with the observation by McCleland et al. that 26% of their 31 triple negative samples stained either 2+ or 3+ for MCT1 by IHC (McCleland et al., 2012). Pre-menopausal patients (relative to their post-menopausal counterparts) also had a higher proportion of cells expressing MCT1, although this may in fact be due to the fact that triple negative disease is more prevalent in pre-menopausal females. Identifying a metabolic signature that is associated with these aggressive features provides an additional insight into this category of breast cancer.

### MCT1 expression is associated with other poor prognostic markers

Triple negative breast cancer is known to be associated with African-American race, younger age at diagnosis, higher clinical stage, higher grade, higher mitotic indices, and pre-menopausal status (Carey et al., 2007). Similarly, MCT1 staining was associated with younger age, higher grade, higher mitotic index, and menopausal status. These are all features of more aggressive disease regardless of their association with triple negative breast cancer. Race and clinical stage were not associated.

We have also investigated histologic subtypes as previous work within triple negative breast cancer has suggested a difference in aggressiveness based upon these characteristics (Brower et al., 1995; Jensen and Page, 2003; Go et al., 2010). With the exception of the solid subtype (−0.11, *p* = 0.008) there were no associations found within our cohort of patients. This is in contrast to other studies showing that the comedo and pure papillary subtypes may be associated with poor prognostic markers and increased invasion on biopsy (Brower et al., 1995; Jensen and Page, 2003; Go et al., 2010). A limitation of this study may be the small numbers of cases of each subtype with the exception of the solid subtype. We also do not have information of gene expression profiling of these tumors in order to determine if gene expression features are associated with MCT1 expression.

There was an association between MCT1 positivity and larger tumor size, suggesting that MCT1 promotes increased carcinoma cell survival and growth (*p* = 0.002). Studies in other cancers have also demonstrated a correlation between high MCT1 staining and advanced tumor stage, particularly in head and neck squamous cell carcinomas (Curry et al., 2013), gastrointestinal cancers (de Oliveira et al., 2012), prostate cancer (Pertega-Gomes et al., 2014), and urothelial cancer (Choi et al., 2014).

### MCT1 expression is associated with poor outcomes

Increased MCT1 staining was associated with a higher recurrence rate with a HR of 2.62 (*p* = 0.024). In addition, the association between a higher percentage of cells staining for MCT1 and death had a hazard ration of 1.89, however this result was not statistically significant (*p* = 0.171). When we investigated whether the associations with higher risk of recurrence was driven by TNBC vs. MCT1 expression, MCT1 expression has an independent contribution to the chance of recurrence.

One potential explanation is that recurrence of disease is more likely when the stroma is metabolically primed to host tumor cells. If chemotherapy failed to eradicate the entirety of the tumor burden, small satellite colonies may more easily settle in soil that has metabolically shifted to serve these tumor cells, thus providing another level of chemo-resistance not previously delineated. To prevent recurrence in high-risk, triple negative disease, it may be necessary to target this soil. MCT1 not only provides diagnostic value but it could serve as an important therapeutic target in the future where chemotherapy would target not only the tumor cell but its stromal energy supply as well. MCT4, a lactate-pyruvate shuttle found in stromal cells, could be MCT1's counterpart therapeutic target. Thus, TNBCs may be further described not only as having a distinct molecular subtype but potentially a distinct metabolic phenotype that can aid in diagnosis and prognostication of this group of tumors.

### Relevance and future directions

The current study is consistent with previous work revealing an association between MCT1 and aggressive breast cancer. MCT1 expression in a TNBC *in vitro* model is associated with cell migration (Gray et al., 2016). Also, Pinheiro et al. have discovered an association between MCT1 expression and up-regulation of basal-associated markers such as CK5, CK14, and vimentin and an inverse relationship of MCT1 to ER and PR expression (Pinheiro et al., 2012). We have discovered that MCT1 is associated with poor outcomes irrespective of breast cancer subtype. MCT1 expression in carcinoma cells may improve risk-stratification of breast cancers. For example, high MCT1 staining may be a marker of a subgroup of very aggressive breast cancers. Inhibitors of MCT1 are in development and may prove to be active in this disease. Our results will need to be evaluated prospectively to confirm the role of MCT1 as a prognostic and predictive biomarker in breast cancer.

## Ethics statement

This study was carried out in accordance with the recommendations of the Declaration of Helsinki. The protocol was approved by the Thomas Jefferson University Institutional Review Committee. Samples were de-identified and no consent was required from participants.

## Author contributions

JJ, PC, RB, and UM were involved in the study design and concept. JJ, PC, RF, LM, JC, DC, MM, DW, MD, ZL, MT, JP, TZ, RB were involved in data acquisition. JJ, PC, RF, LM, JC, DC, TZ, MT, JP, RB, and UM were involved in data analysis and interpretation. JJ, RF, LM, and JC drafted the manuscript. JJ and UM edited the manuscript.

## Funding

The National Cancer Institute of the National Institutes of Health under Award Numbers K08 CA175193 and P30CA056036 supported this work. Funding was used to provide material support for laboratory testing.

### Conflict of interest statement

The authors declare that the research was conducted in the absence of any commercial or financial relationships that could be construed as a potential conflict of interest.

## Abbreviations

MCT: monocarboxylate transporter
TNBC: triple negative breast cancer
ER: estrogen receptor
PR: progesterone receptor
BLBC: basal like breast cancer
CK: cytokeratin
ATP: adenosine triphosphate
GIST: gastrointestinal stromal tumor
AJCC: American Joint Committee on Cancer
ISH: *in-situ* hybridization.
